# Melatonin Modulation of Sirtuin-1 Attenuates Liver Injury in a Hypercholesterolemic Mouse Model

**DOI:** 10.1155/2018/7968452

**Published:** 2018-02-04

**Authors:** Francesca Bonomini, Gaia Favero, Luigi Fabrizio Rodella, Mohammed H. Moghadasian, Rita Rezzani

**Affiliations:** ^1^Anatomy and Physiopathology Division, Department of Clinical and Experimental Sciences, University of Brescia, Viale Europa 11, 25123 Brescia, Italy; ^2^Interdipartimental University Center of Research “Adaption and Regeneration of Tissues and Organs (ARTO)”, University of Brescia, Viale Europa 11, 25123 Brescia, Italy; ^3^Department of Human Nutritional Sciences, University of Manitoba and Canadian Centre for Agri-Food Research in Health and Medicine, St. Boniface Hospital Research Centre, Winnipeg, MB, Canada

## Abstract

Hypercholesterolemia increases and exacerbates stress signals leading also to liver damage (LD) and failure. Sirtuin1 (SIRT1) is involved in lifespan extension and it plays an essential role in hepatic lipid metabolism. However, its involvement in liver hypercholesterolemic damage is not yet completely defined. This* in vivo* study evaluated the role of SIRT1 in the hypercholesterolemic-related LD and, then, investigated how oral supplementation of melatonin, pleiotropic indoleamine, may be protective. Control mice and apolipoprotein E-deficient mice (ApoE^−/−^) of 6 and 15 weeks of age were treated or not treated with melatonin at the dose of 10 mg/kg/day for 9 weeks. In this study, we evaluated serum biochemical markers, liver SIRT1 expression, and oxidative stress markers. We observed that hypercholesterolemia increased significantly serum cholesterol and triglycerides, reduced significantly liver SIRT1, and, in turn, induced hepatic oxidative stress in untreated ApoE^−/−^ mice with respect to control mice. Interestingly, melatonin treatment improved serum biochemical markers and hepatic morphological impairment and inhibited oxidative stress through its antioxidant properties and also by SIRT1 upregulation. In summary, melatonin oral supplementation may represent a new protective approach to block hypercholesterolemic liver alterations involving also a SIRT1-dependent mechanism.

## 1. Introduction

Hyperlipidemia, which included hypercholesterolemia (HC), is the most striking risk factor in the development of cardiovascular diseases in occidental population [1]. Besides its correlation with cardiovascular events, HC is considered a risk factor that contributes to liver damage (LD). Moreover, this disorder has been reported in 20%–80% of cases of nonalcoholic fatty liver disease (NAFLD) [2] and may induce liver inflammatory cell infiltration, fibrosis, and production of chemokines, cytokines, and oxidative factors that, in turn, lead to oxidative stress (OS) and inflammation [3–5]. OS, mitochondrial dysfunction, and upregulation of proinflammatory cytokines have been suggested to be the major consequences of cellular lipid overload [6, 7], contributing, in turn, to hepatic inflammatory damage and fibrogenesis [8, 9]. Moreover, OS characterized by decreased activity of endogenous antioxidant system, with a reduction of superoxide dismutase1 (SOD1) and catalase (CAT), together with an increase in inducible nitric oxide synthase (iNOS), appears to be responsible for lipid-related LD in different experimental models [10–12]. This is consistent with previous findings from our and other research groups showing that hypercholesterolemic mice have important morphological changes at liver level, like disorganization of hepatic parenchyma with widespread cell swelling, congestion of sinusoids, hepatocytes fatty deposits, polyploidy, and augmented nuclear size [13, 14].

Recently, it has been reported that sirtuins, belonging to silent information regulator 2 family, play a key role in the development and rescue of various metabolic diseases, including NAFLD [15–17]. In fact, sirtuin1 (SIRT1) is involved in the prevention of the development of NAFLD through its role in the regulation of inflammation and lipid metabolism. SIRT1 regulates lipid metabolism through both its deacetylase activity and its direct and indirect involvement in insulin signaling [18]. Furthermore, SIRT1 modulates activities of many hepatic gene-regulatory proteins and it may activate gluconeogenesis and fatty acid oxidation or induce the lipogenesis inhibition in experimental conditions [19, 20]. Liver-specific deletion of SIRT1, as well as protein downregulation, resulted in hepatic steatosis, inflammation, and OS [21, 22].

It is known that the inhibition of SIRT1 signaling in human fetal hepatocytes induces an increase in intracellular glucose and lipid levels with upregulation of de novo lipogenesis and gluconeogenesis related genes [23]. Moreover, the hepatic deletion of SIRT1, as well as SIRT1 downregulation, caused hepatic steatosis and inflammation [24].

Currently available therapies for controlling hyperlipidemia, such as fibrates, bile acid sequestraints, and statins, are almost inefficient in lipid metabolism regulation and cause different side effects in patients [25]. Worldwide efforts aimed at reducing the impact of hyperlipidemia are among the top priorities and, to date, several strategies including nutritional supplements and anti-inflammatory treatments have been suggested to counteract HC [26].

Melatonin is an indoleamine produced and secreted into blood stream during dark period by the pineal gland. Its physiological effects include regulation of seasonal reproduction, body weight, and energy balance [27, 28]. Furthermore, melatonin acts as a highly effective antioxidant and anti-inflammatory molecule [29] with beneficial actions also against obesity and related LD [30, 31]. Further, it is known that melatonin increases, in different experimental conditions, the hepatic level of SIRT1 as well as in vessels of apolipoprotein E-deficient mice (ApoE^−/−^) [32, 33].

Since the mechanisms involved in HC-related LD are not fully understood, the aim of this study was to better investigate liver morphological alterations during HC. Then, we evaluated if melatonin is effective in reducing LD and OS through also the induction of SIRT1 expression. To address this issue, we used ApoE^−/−^ mice, an animal model that spontaneously develops HC, aortic lipid accumulation, and LD in a time-dependent manner. As previously shown, ApoE^−/−^ mice develop HC from the 4th week of life and then showed alterations in hepatic morphology and protein expressions [34–37].

In the present study, we provided evidence that melatonin restores liver cytoarchitecture preventing SIRT1 reduction. Furthermore, SIRT1 is able to decrease OS inducing also antioxidants expression. Then, we suggested that melatonin may be a valuable protective alternative strategy for minimizing the LD related to HC, via also a SIRT1-dependent mechanism of action.

## 2. Materials and Methods

### 2.1. Animal Treatment

Forty C57BL/6 male mice and thirty ApoE^−/−^ male mice (Harlan Laboratories S.r.l., Udine, Italy and Charles River Laboratories S.r.l., Lecco, Italy) were housed in an animal experimental unit with 12 h alternating light–dark cycle and constant temperature. Animal had free access to food and water. All the protocols were approved by the Italian Ministry of Health and complied with “Guiding Principles in the Use of Animals in Toxicology,” which were adopted by the Society of Toxicology in 1989.

Mice were randomly divided into the following seven groups (ten animals per group): (1) control untreated C57BL/6 mice which were 6 weeks old at sacrifice; (2) control untreated C57BL/6 mice which were 15 weeks old at sacrifice; (3) control C57BL/6 mice treated orally with 1% ethanol (melatonin vehicle) dissolved in tap water from the 6th to 15th week of life; (4) control C57BL/6 mice treated orally with melatonin (10 mg/kg/day) from the 6th to 15th week of life; (5) ApoE^−/−^ untreated mice which were 6 weeks old at sacrifice (ApoE^−/−^ of 6 w); (6) ApoE^−/−^ untreated mice which were 15 weeks old at sacrifice (ApoE^−/−^ of 15 w); and (7) ApoE^−/−^ mice treated orally with melatonin (10 mg/kg/day) from the 6th to 15th week of life (ApoE^−/−^ + MEL).

Animals were individually housed in cages with a single water bottle to ensure that all received the correct melatonin dose according to the body weight of the animal. The bottles were wrapped in aluminium foil to protect melatonin from light. Synthetic melatonin was dissolved in 1% ethanol and then diluted in tap water to yield a final dose of 10 mg/kg body weight/day, as previously described by Rezzani et al. [38].

At the end of the study, all the animals were euthanized and blood and liver samples were collected. Serum cholesterol and triglyceride concentrations were determined by standard laboratory procedures and also serum SOD1 and glutathione (GSH) levels were evaluated, as described later. Furthermore, liver samples were weighted and then fixed in 4% paraformaldehyde and embedded in paraffin wax, according to standard procedures [38, 39]. Serial sections were cut using a microtome (7 *μ*m thickness) for the following described morphological, immunohistochemical/immunofluorescence, and histomorphometrical analyses.

### 2.2. Haematoxylin-Eosin Staining

Liver paraffin-embedded sections were deparaffinized, rehydrated, and then stained with haematoxylin-eosin, following standard protocols. In detail, haematoxylin-eosin staining was used to evaluate liver morphology and also to measure the hepatocyte nuclear area [13, 40]. The hepatocyte nuclear area was calculated using a computerized image analyzer (Image Pro Premier 9.1, Media Cybernetics Inc., Rockville, USA) evaluating 20 randomly chosen liver fields per experimental animal. Two blinded investigators performed the morphometrical analysis and their evaluation was assumed to be correct if the values were not significantly different. If there was disagreement concerning the interpretation, the case was reconsidered to reach a unanimous agreement.

### 2.3. Picrosirius Red Staining

Picrosirius red staining was used to evaluate liver fibrosis [41]. In detail, paraffin-embedded liver sections, after deparaffinization and rehydration, were stained for 5 minutes in 1% phosphomolybdic acid aqueous solution and then for 3 minutes in 6% Sirius red aqueous solution. Polarized light allowed visualization of collagen fibers of different thickness with different colours, as previously described [34, 42].

### 2.4. Immunohistochemistry and Double Immunofluorescence Analyses

Liver paraffin-embedded sections were deparaffinized, rehydrated, and incubated for 1 hour at room temperature in specific normal serum. Subsequently, the sections were incubated overnight at 4°C with the following primary antibodies: rabbit polyclonal antibody against SIRT1 (diluted 1 : 100; Santa Cruz Biotechnology, Santa Cruz, CA, USA) or rabbit polyclonal antibody against iNOS (diluted 1 : 200; Santa Cruz Biotechnology, Santa Cruz, CA, USA) or simultaneously with goat antibody against SOD1 (diluted 1 : 100; Santa Cruz Biotechnology, Santa Cruz, CA, US) and rabbit polyclonal antibody against CAT (diluted 1 : 150; Santa Cruz Biotechnology, Santa Cruz, CA, US). Liver sections were then washed in tris-buffered saline (TBS) and, for immunohistochemical analysis of SIRT1 and iNOS, were incubated for 1 hour with specific biotinylated secondary antibody and after 1 hour with the avidin-biotin horseradish peroxidase complex (ABC-peroxidase kit Vector Labs, Burlingame, CA, USA), prepared according to the manufacturer's instructions. Finally, these liver sections were immersed in a solution of 0.05% 3′-3′-diaminobenzidine tetrahydrochloride (DAB) and 0.03% hydrogen peroxide for 10 minutes, counterstained with haematoxylin, dehydrated, mounted, and observed with a light microscopy (Olympus, Hamburg, Germany) at a final magnification of 400x.

However, for the double immunofluorescence staining with SOD1 and CAT, the liver sections, after the incubation with primary antibodies, were labelled using anti-goat Alexa Fluor 546 and anti-rabbit Alexa Fluor 488 conjugated secondary antibodies (diluted 1 : 200; Invitrogen, UK). Finally, the sections were counterstained with 4′-6-diamidino-2-phenylindole (DAPI), mounted, and observed with a confocal microscope (510 Meta Zeiss, Munich, Germany) at a final magnification of 400x, as previously described by Agabiti-Rosei et al. [43].

Control reactions for both immunohistochemistry and immunofluorescence analysis were performed in absence of primary antibody and in the presence of isotype-matched IgGs.

Randomly chosen 20 liver fields for each experimental animal were analyzed and the immunostaining for each primary antibody was calculated using an image analyzer (Image Pro Premier 9.1, Media Cybernetics Inc., Rockville, USA). Two blinded investigators performed the histomorphometrical analysis and their evaluation was assumed to be correct if the values were not significantly different. If there was disagreement concerning the interpretation, the case was reconsidered to reach a unanimous agreement. The levels of immunostaining of each primary antibody evaluated were expressed as arbitrary units (AU).

### 2.5. Measurement of Serum Levels of Superoxide Dismutase1 and Glutathione

Serum samples were obtained collecting total blood in serum separator tubes and allowed samples to clot for 2 hours at room temperature. Then the samples were centrifuged for 20 minutes at 1000 ×g. The supernatants obtained were subjected to analyses of the levels of SOD1 and GSH through specific ELISA assay kits and following the respective manufacturer's instructions (LifeSpan BioSciences, Inc., Seattle, USA). In detail, the optical density values for both SOD1 and GSH were determined using a microplate reader set at 450 nm. Furthermore, SOD1 ELISA assay presented intra-assay variations coefficient less than 4.7%, interassay variations coefficient less than 9.5%, and sensitivity less than 1.56 U/mL, whereas the GSH ELISA assay had intra-assay variations coefficient less than 6.2%, interassay variations coefficient less than 9.6%, and sensitivity less than 1 ng/mL.

### 2.6. Statistical Analysis

The data are expressed as mean ± SD. All data were analyzed by one-way analyses of variance for repeated measures (ANOVA), corrected by Bonferroni, to compare the variability of a group with all other experimental groups. Probability values less than 0.05 were considered significant. All experiments were carried out in triplicate and data were collected and analyzed by Origin Pro 9.1 software (OriginLab Corporation, Northampton, MA, USA).

## 3. Results

### 3.1. Animal Treatment and Serum Biochemistry

The animal body weight measured before the start and at different time points of the treatment and also the liver weight measured at the end of the study did not show any significant difference among the experimental groups.  Table 1 summarized the percentage of liver weight respect body weight.

As expected, ApoE^−/−^ 6 w and 15 w had increased serum cholesterol and triglyceride levels compared to control mice. In particular, both serum cholesterol and triglyceride concentrations were higher in ApoE^−/−^ 15 w with respect to ApoE^−/−^ 6 w. Interestingly, both serum parameters were significantly reduced after melatonin treatment of ApoE^−/−^ mice (Table 1).

### 3.2. Haematoxylin-Eosin Staining

Haematoxylin-eosin staining showed that in untreated control C57BL/6 mice which were 6 weeks or 15 weeks old or in control C57BL/6 mice treated with 1% ethanol (vehicle of melatonin) or with melatonin presented a “normal” hepatic parenchyma without LD. These observations underline that all the above reported experimental groups are similar, so they are considered a single group defined generically as “control” in the following morphological, immunohistochemical/immunofluorescence, and histomorphometrical analysis.

**Figure 1:**
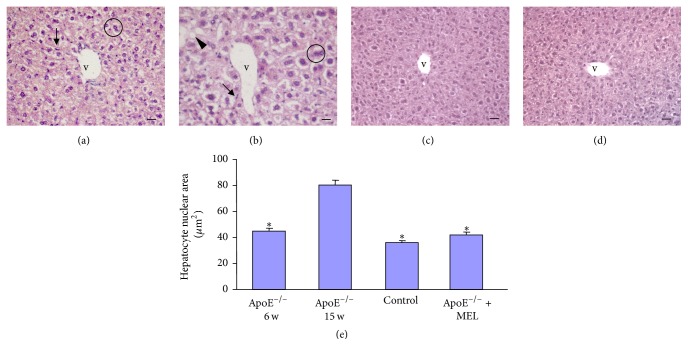
Photomicrographs of liver haematoxylin-eosin staining of untreated ApoE^−/−^ of 6 w (a), untreated ApoE^−/−^ of 15 w (b), control (c), and ApoE^−/−^ + MEL (d). Bar: 20 *μ*m. v: central vein, the arrowhead indicates lipid droplet deposition, the arrow identifies the inflammatory cells infiltration, and the circle indicates hepatocyte nuclear polyploidy. The graph (e) summarized the morphometrical analysis of hepatocyte nuclear area. ^**∗**^*p* ≤ 0.05 versus ApoE^−/−^ of 15 w.

In both untreated ApoE^−/−^ 6 w and 15 w groups, we observed significant LD. In fact, both untreated groups presented hepatocyte ballooning, nuclear polyploidy, and granular cytoplasm with prevalently small lipid droplets deposition, characteristic of microvesicular steatosis, together with inflammatory cells infiltration. The reported hepatic parenchymal alterations were weak/moderate in ApoE^−/−^ 6 w (Figure 1(a)) and moderate/strong in ApoE^−/−^ 15 w (Figure 1(b)), with respect to liver of control mice that showed “normal” hepatic parenchyma with radially arranged rays of hepatocytes, regularly directed from the central vein of each lobule towards its periphery (Figure 1(c)). Liver lipid droplets deposition, hepatocyte ballooning, nuclear polyploidy, and inflammatory infiltration were significantly decreased in liver of ApoE^−/−^ + MEL, exhibiting an almost “normal” hepatic cytoarchitecture (Figure 1(d)).

Furthermore, untreated ApoE^−/−^ 15 w showed a significant increased hepatocyte nuclear area with respect to both ApoE^−/−^ 6 w and control groups. Remarkably, liver of ApoE^−/−^ + MEL exhibited a significant hepatocyte nuclear area reduction, reaching value comparable to liver of control mice (Figure 1(e)).

### 3.3. Picrosirius Red Staining

**Figure 2:**
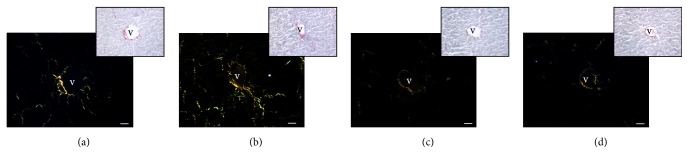
Photomicrographs of liver Picrosirius red staining of untreated ApoE^−/−^ of 6 w (a), untreated ApoE^−/−^ of 15 w (b), control (c), and ApoE^−/−^ + MEL (d) under polarized light (a–d) or without polarized light (inserts). Bar: 20 *μ*m. v: central vein.

Picrosirius red staining showed a mild parenchymal hepatic perisinusoidal fibrosis in untreated ApoE^−/−^ 6 w (Figure 2(a)) and a moderate/strong perisinusoidal collagen deposition in liver of untreated ApoE^−/−^ 15 w (Figure 2(b)) with respect to liver of control mice that showed very weak parenchymal fibrosis (Figure 2(c)). The liver of ApoE^−/−^ + MEL exhibited a significant fibrosis reduction (very weak collagen deposition) (Figure 2(d)).

### 3.4. Immunohistochemical Analysis of Sirtuin1

**Figure 3:**
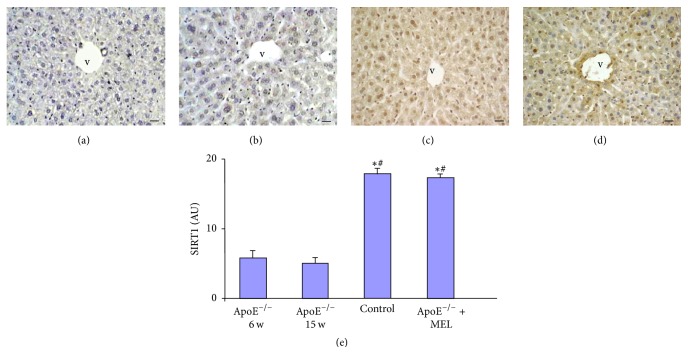
Photomicrographs of liver SIRT1 immunohistochemical analysis of ApoE^−/−^ of 6 w (a), ApoE^−/−^ of 15 w (b), control (c), and ApoE^−/−^ + MEL (d). Bar: 20 *μ*m. v: central vein. The graph (e) summarizes the SIRT1 histomorphometrical analysis. ^**∗**^*p* ≤ 0.05 versus ApoE^−/−^ of 6 w and ^#^*p* ≤ 0.05 versus ApoE^−/−^ of 15 w.

Immunohistochemical analysis of SIRT1 showed a very weak/weak expression at parenchymal liver level of both untreated ApoE^−/−^ 6 w (Figure 3(a)) and untreated ApoE^−/−^ 15 w (Figure 3(b)), relative to liver of control mice that exhibited diffuse and strong expression at both hepatocytes cytoplasm and nuclear level (Figure 3(c)). The liver of ApoE^−/−^ + MEL exhibited a significant increase in SIRT1 expression at both cytoplasm and nuclear levels (Figure 3(d)), reaching level almost comparable to liver of control mice.

These observations were confirmed also by liver SIRT1 histomorphometrical analysis (Figure 3(e)).

### 3.5. Immunohistochemical Analysis of Oxidative Stress Marker (iNOS) and Double Immunofluorescence Analysis of Antioxidants (SOD1 and CAT)

**Figure 4:**
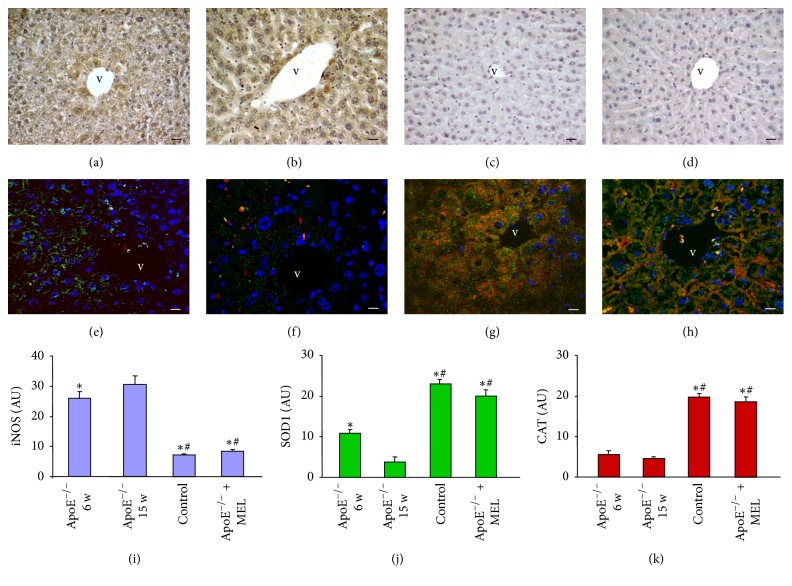
Photomicrographs of liver immunohistochemical analysis of iNOS (a–d) and of liver double immunofluorescence analysis of SOD1 (green staining) and CAT (red staining) (e–h) of ApoE^−/−^ of 6 w (a, e), ApoE^−/−^ of 15 w (b, f), control (c, g), and ApoE^−/−^ + MEL (d, h). Bar: 20 *μ*m. v: central vein. The graphs summarized the iNOS (i), SOD1 (j), and CAT (k) histomorphometrical analysis. ^**∗**^*p* ≤ 0.05 versus ApoE^−/−^ of 6 w and ^#^*p* ≤ 0.05 versus ApoE^−/−^ of 15 w.

Both untreated ApoE^−/−^ 6 w and 15 w groups showed a significant iNOS expression at parenchymal liver level; in particular, liver of untreated ApoE^−/−^ 6 w had a moderate iNOS expression (Figure 4(a)) and liver of untreated ApoE^−/−^ 15 w exhibited a strong expression (Figure 4(b)), relative to liver of control mice that showed very weak/weak iNOS expression (Figure 4(c)). Remarkably, liver of ApoE^−/−^ + MEL showed a significant reduction of iNOS expression (Figure 4(d)), similar to liver of control mice. The hepatic iNOS expression was localized prevalently at hepatocyte cytoplasm throughout the hepatic lobule.

The double immunofluorescence evaluation of endogenous antioxidants SOD1 (identified in green staining) and CAT (identified in red staining) showed that parenchymal liver of untreated ApoE^−/−^ 6 w had a mild hepatic expression of SOD1 and a very weak/weak CAT expression (Figure 4(e)). Instead, liver of untreated ApoE^−/−^ 15 w showed very weak/weak expression of both antioxidants (Figure 4(f)) with respect to liver of control mice that had diffuse and strong parenchymal expression of both SOD1 and CAT (Figure 4(g)). Interestingly, liver of ApoE^−/−^ + MEL showed a significant increase of both antioxidants (Figure 4(h)), reaching values comparable to liver of control mice. Both antioxidants were expressed at hepatocyte cytoplasm level and no nuclear positivity was observed.

All the above reported observations were confirmed also by the histomorphometrical hepatic analysis of iNOS (Figure 4(i)), SOD1 (Figure 4(j)), and CAT (Figure 4(k)).

### 3.6. Serum Levels of Superoxide Dismutase1 and Glutathione

**Figure 5:**
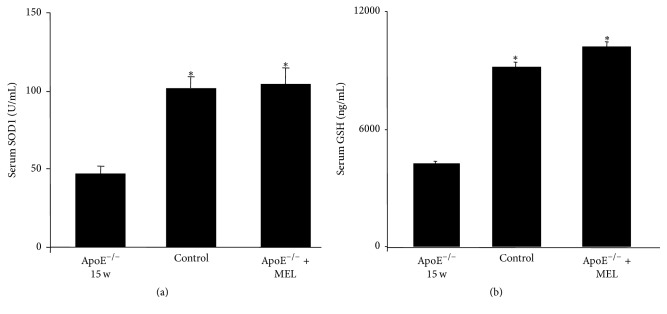
Serum superoxide dismutase1 (SOD1) (a) and glutathione (GSH) (b) levels. ^*∗*^*p* ≤ 0.05 versus ApoE^−/−^ of 15 w.

Next to histomorphometrical analyses of SOD1 and CAT, we evaluated also the serum levels of the antioxidant enzymes SOD1 and GSH. ApoE^−/−^ 15 w showed a significant reduction of both SOD1 and GSH levels with respect to control mice that showed strong/moderate serum levels. Remarkably, melatonin treatment of ApoE^−/−^ mice showed a significant increase of both serum antioxidants (strong levels).  Figure 5(a) summarized SOD1 serum levels, whereas GSH serum levels are represented in Figure 5(b).

## 4. Discussion

The LD observed in an ApoE^−/−^ hypercholesterolemic mouse model were significantly reduced by oral supplementation of melatonin.

We observed a severe decrease of SIRT1 expression in liver of ApoE^−/−^ mice, more evident at 15 weeks of life. Growing evidences from both human and experimental studies revealed that impaired SIRT1 signaling is associated with alcoholic liver disease and genetic or pharmacological stimulations of SIRT1 protect against steatosis and/or steatohepatitis [44, 45]. Furthermore, liver-specific SIRT1 deficiency is related to hepatic glucose overproduction, chronic hyperglycemia, and increased OS [12, 22].

To highlight the importance of hepatic SIRT1 expression and its involvement in OS, we evaluated the expression of iNOS, a proinflammatory protein commonly related to OS status, and of endogenous antioxidants (SOD1, CAT, and GSH). Our results showed that iNOS is strongly expressed in liver of ApoE^−/−^ mice and this increase plays, in turn, a critical role in the development of liver inflammation and OS [46, 47]. We observed also a significant low expression of antioxidant enzymes in ApoE^−/−^ 15 w both in hepatic parenchyma and at serum level. It is known that, under physiological conditions, these enzymes can efficiently counteract oxidative damage induced by free radicals, while in pathological conditions there are no enough endogenous antioxidants to counteract the excessive free radicals [48].

Remarkably, our results exhibited that the oral supplementation of melatonin restored SIRT1 hepatic expression that, in turn, had an antagonistic effect to LD and OS in ApoE^−/−^ mice. These findings confirmed the literature data, reporting that melatonin had important antioxidant effects and also a strong tendency to attenuate hepatic steatosis [49]. In our opinion, the protective effects of melatonin against LD related to HC may be attributed not only to its direct and/or indirect antioxidant properties, but probably also through its action mediated by SIRT1 that is involved in the inhibition of iNOS and preservation of the endogenous antioxidant system. Moreover, our results are in agreement with Lee et al. [50] who showed that treatment of a *β*-cell line (RIN) with cytokines induced cell damage correlated with the increased iNOS expression. However, SIRT1 overexpression completely prevented cytotoxicity and the increase of iNOS by the inhibition of the nuclear factor-kB signaling. Tanno et al. [51] also showed that SIRT1 participate in the modulation of the endogenous antioxidants.

Other experimental studies showed that melatonin alone or administered in combination with pioglitazone or pentoxifylline reduced the insulin resistance index and total cholesterol and triglycerides and modulated GSH level during NAFLD [52].

## 5. Conclusion

**Figure 6:**
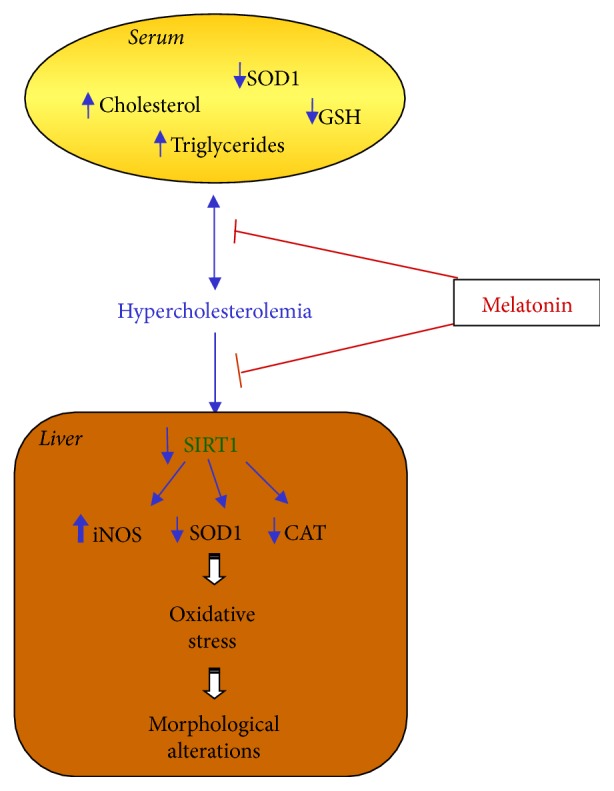
Schematic representation of melatonin effects against hypercholesterolemic hepatic alterations. It is important to underline that this indoleamine, together with its antioxidant properties, is able to reduce liver morphological alterations also blocking the decrease of sirtuin1 that in turn inhibits oxidative stress. The red symbols indicate the effect of melatonin in blocking the pathways induced by hypercholesterolemia that are indicated by the blue arrows. CAT: catalase; GSH: glutathione; iNOS: inducible nitric oxide synthase; SIRT1: sirtuin1; SOD1: superoxide dismutase1.

Our results suggest that oral supplementation of melatonin inhibits LD induced by HC and that not only could this effect be due to its known antioxidant properties, but also it may be mediated by a SIRT1-dependent mechanism that, in turn, blocks serum cholesterol and triglycerides increase and hepatic iNOS expression and restores endogenous antioxidants (Figure 6). Further studies are needed for addressing the involvement of SIRT1 in HC and the mechanism(s) by which melatonin counteracts LD.

## Figures and Tables

**Table 1:** Liver/body weight ratio and serum parameters.

	ApoE^−/−^ 6 weeks	ApoE^−/−^ 15 weeks	Control 6 weeks	Control 15 weeks	ApoE^−/−^ plus melatonin 15 weeks
Liver/body weights (%)	5.4	5.5	5	5.2	5.1
Cholesterol (mg/dL)	216.5 ± 18.7	266.2 ± 15^*∗*^	75.4 ± 11.7^*∗*#^	111.8 ± 12.6^*∗*#+^	153.8 ± 14.7^*∗*#+°^
Triglycerides (mg/dL)	56.3 ± 4.5	58.2 ± 2.6	11 ± 0.6^*∗*#^	16.3 ± 1.2^*∗*#^	37.9 ± 6.4^*∗*#+°^

^*∗*^ *p* ≤ 0.05 versus ApoE^−/−^ (6 weeks old); ^#^*p* ≤ 0.05 versus ApoE^−/−^ (15 weeks old); ^+^*p* ≤ 0.05 versus control mice (6 weeks old);  °*p* ≤ 0.05 versus control mice (15 weeks old).
